# Assessing Apoptosis Gene Expression Profiling with a PCR Array in the Hippocampus of Ts65Dn Mice

**DOI:** 10.1155/2015/214618

**Published:** 2015-05-05

**Authors:** Bin Yu, Bin Zhang, Wen-bo Zhou, Qiu-wei Wang, Pei Yuan, Yu-qi Yang, Jing Kong

**Affiliations:** Changzhou Woman and Children Health Hospital Affiliated to Nanjing Medical University, No. 26 Bo Ai Road, Changzhou, Jiangsu 213003, Jiangsu Province, China

## Abstract

It is well known that Down syndrome (DS) is a condition in which extra genetic material causes delays in the way a child develops, both mentally and physically. Intellectual disability is the foremost and most debilitating trait, which caused loss of cognitive abilities and the development of early onset Alzheimer's disease (AD). Ts65Dn mice were used in this study. We isolated the hippocampus. First, we used transmission scanning electron microscopy to directly observe the hippocampus and confirm if apoptosis had occurred. Second, we customized a PCR array with 53 genes, including several important genes related to cell apoptosis. Gene expression was detected by RT-PCR. There were varying degrees of changes characteristic of apoptosis in the hippocampus of Ts65Dn mice, which mainly included the following: nuclear membrane thinning, unevenly distributed chromosomes, the production of chromatin crescents, and pyknosis of the nuclei with some nuclear fragmentation. Meanwhile, three genes (API5, AIFM1, and NF*κ*B1) showed changes of expression in the hippocampus of Ts65Dn mice compared with normal mice. Only NF*κ*B1 expression was significantly increased, while the expressions of API5 and AIFM1 were notably decreased. The fold changes in the expression of API5, AIFM1, and NF*κ*B1 were 11.55, 5.94, and 3.11, respectively. However, some well-known genes related to cell apoptosis, such as the caspase family, Bcl-2, Bad, Bid, Fas, and TNF, did not show changes in expression levels. The genes we found which were differentially expressed in the hippocampus of Ts65Dn mice may be closely related to cell apoptosis. PCR array technology can assist in the screening and identification of genes involved in apoptosis.

## 1. Introduction

Down syndrome (DS) is one of the most common gross chromosomal abnormalities and birth defects. It is well known that DS is a condition in which extra genetic material causes delays in child development, both mentally and physically. Intellectual disabilities are the most debilitating trait and they cause the loss of cognitive abilities. Meanwhile, it is well known that Alzheimer's disease occurs in individuals with trisomy 21 because the gene encoding amyloid is located on this chromosome. Thus, these individuals have a higher load of amyloid which, in turn, results in a higher incidence of developing cognitive decline and Alzheimer's dementia with aging [1]. Language abilities, learning, and memory are impaired in DS patients, which restricts their life and social skills. Therefore, increasing our knowledge of the neural defects associated with DS is of great significance.

Apoptosis is the mechanism by which cells die in response to a wide range of physiological and developmental stimuli. Increasing data has shown that enhanced apoptosis in Alzheimer's disease might play a role in the mental deficiencies and neurodegeneration associated with that disease [2, 3]. However, little is known about apoptotic processes associated with DS, and the existing results concerning this issue are inconsistent. Current reports regarding apoptosis in DS have focused on the apoptosis-related proteins. Engidawork's group [4] reported that BIM/BOD, Bcl-2, and p21 were significantly increased in the frontal cortex and cerebellum of DS patients, and no detectable changes were obtained in the expression of Fas, caspase-3, and annexins (I, II, V, and VI) compared to controls [5]. Akira [6] also reported the downregulation of Bcl-2 and Bcl-x mRNA in DS patients. Both findings provide further evidence that apoptosis indeed accounts for the neuronal loss in DS. Additionally, the disruption of neurogenesis and apoptosis, which are the two fundamental processes underlying brain formation, has been reported, which could reduce the number of neurons in the DS hippocampal region [7]. However, little is known about apoptotic processes in DS patients, and the results are contradictory. Some studies have shown increases in the number of apoptotic cells in human DS brains [7] as well as Ts65Dn mice [8] and Ts1Cje cells [9]. However, some groups have demonstrated no differences or even a reduced amount of apoptotic cell death in DS human and Ts65Dn mouse brains [10]. In short, there is no conclusion as to whether increased apoptosis occurs in the nervous system of Down syndrome patients.

Down syndrome results from the presence of an extra chromosome 21, which causes changes in gene expression. It is not known whether this unbalanced gene regulation is related to apoptosis in the nervous system. However, there are so many molecules involved in the process of apoptosis that real-time quantitative PCR may not be suitable for observing it. A high-throughput platform with efficient detection capacity is needed to solve this problem. Recently, PCR arrays have been considered the most reliable tools for analyzing the expression of a focused panel of genes, especially in signal transduction pathways, biological process, and disease related gene networks [11]. PCR arrays have been used for cancer, immunology, stem cells, toxicology, and many other areas of biological and medical research.

In present study, we directly observed whether apoptosis occurred in the nervous system of Ts65Dn mice with a transmission scanning electron microscope. Meanwhile, a target PCR array related to apoptosis was constructed and applied to screen for changes in expression of genes related to apoptosis.

## 2. Materials and Methods

This study was conducted in the Changzhou Women and Children Hospital of Nanjing Medical University (Changzhou, Jiangsu, China). The animals were bred in the Animal Center of Jiangsu University (Zhenjiang, Jiangsu, China). All efforts were made to minimize the suffering and number of animals used.

### 2.1. Animals

Five Ts65Dn mice carrying a partial trisomy of chromosome 16 were purchased from the Jackson Laboratories (Bar Harbor, ME, USA). Five normal C57BL/6JEi mice were used as the normal control group. Both groups were matched by age and were more than 14 weeks old. The animals' health and comfort were monitored by the veterinary service. The animals had access to water and food according to the routine methods of animal breeding in the Animal Center of Jiangsu University.

### 2.2. Methods

#### 2.2.1. Sample Collection

Anesthetized animals were euthanized using 10% chloral hydrate (Changzhou First People's Hospital, Changzhou, China). We removed the brain and isolated the hippocampus.

#### 2.2.2. Transmission Electron Microscopy

To assess the morphological changes in the hippocampus of Ts65Dn mice, we used transmission scanning electron microscopy to directly observe whether apoptosis had occurred. The methods were performed as described by Chen's group [12]. Briefly, the hippocampal tissues were perfused with 4% paraformaldehyde. After sufficient washing with 0.1 M PB, the tissues were postfixed with 6% osmium tetroxide for 2 h. Then, they were rinsed with distilled water before undergoing a graded ethanol dehydration series. After they were infiltrated with a mixture of one-half acetone and one-half resin for 2 h, the tissues were polymerized in resin for 12 h. After four days, the tissues were embedded in resin and were cut into suitable squares. Then, they were stained with 4% uranyl acetate. Finally, sections from each hippocampus were observed under a transmission electron microscope (CM100; PHILIPS, Amsterdam, Holland) (maximal magnification 1 × 450000, resolution 0.34 nm).

#### 2.2.3. Total RNA Extraction

Total RNA was isolated by TRIZOL (Invitrogen) extraction. After the homogenization of tissue samples, insoluble material was removed from the homogenate by centrifugation at 12000 ×g for 10 minutes at 2 to 8°C. The homogenized samples were incubated for 5 minutes at room temperature, and 0.2 mL of chloroform per 1 mL of TRIZOL reagent was added. The tubes were shaken vigorously by hand for 15 seconds and incubated at 15 to 30°C for 2 to 3 minutes. Then, the samples were centrifuged at no more than 12000 ×g for 15 minutes at 2 to 8°C, and the colorless upper aqueous phase was transferred to a fresh tube. The RNA was precipitated from the aqueous phase by mixing with isopropyl alcohol (0.5 mL of isopropyl alcohol per 1 mL of TRIZOL reagent). Samples were incubated at 15 to 30°C for 10 minutes and centrifuged at no more than 12000 ×g for 10 minutes at 2 to 8°C. The supernatant was removed, and the RNA pellet was washed once with 75% ethanol. The samples were mixed by vortexing and centrifuged at no more than 7500 ×g for 5 minutes at 2 to 8°C. Finally, the RNA pellets were air or vacuum dried for 5–10 minutes, and RNA was dissolved in RNase-free water.

#### 2.2.4. PCR Array Customization

PCR arrays contained 53 genes, 6 housekeeping genes, PPC, and GDC. The 53 genes were related to cell apoptosis. The housekeeping genes were B2M, ACTB, GAPDH, RPL27, HPRT1, and OAZ1. The PPC contained synthetic DNA fragments that have no homology with the detected species and amplification primers which were used as quality control. GDC was used to detect the residual genomic DNA.

#### 2.2.5. PCR Array Experiment

Total RNA from each sample was used for reverse transcription with an RT-PCR Kit (catalog#CTB101; CT biosciences, China) on an ABI 9700 thermocycler (ABI, Foster City, CA). For reverse transcription, 4 *μ*L of total RNA was mixed with 10 *μ*L of OligodT Primer (10 *μ*M), and the solution was incubated at 70°C for 10 min and then quickly cooled on ice for 2 min. The cooled solution was mixed with 4 *μ*L of 5 × reverse transcription buffer, 1 *μ*L of dNTP (10 mM), 0.5 *μ*L of RNasin (40 U/*μ*L), and 0.5 *μ*L of reverse transcriptase (200 U/*μ*L). Reverse transcription was performed at 42°C for 1 hour, followed by an inactivation reaction at 70°C for 15 min. The resulting cDNA was stored at −20°C until used. PCR arrays were performed with customized PCR containing predispensed primers (CT biosciences, China) on a LightCycler 480 (Roche Diagnostics, Mannheim, Germany) using SYBR Master Mix (catalog#CTB101; CT biosciences, China). The thermocycler parameters consisted of an initial denaturation at 95°C for 2 min followed by 45 cycles of denaturation at 95°C for 10 s and annealing at 60°C for 20 s. Relative changes in gene expression were calculated using the ΔΔCt (threshold cycle) method. The housekeeping genes (B2M, ACTB, GAPDH, RPL27, HPRT1, and OAZ1) were used to normalize the amount of RNA. Fold change values were calculated using the 2-ΔΔCt formula.

### 2.3. Statistical Analysis

All data were collected and statistically analyzed using SPSS 13.0 software. The results were expressed as the means ± SD. The expression of genes in two groups was compared using *t*-tests. A *P* value < 0.05 was considered statistically significant.

## 3. Results

### 3.1. Apoptosis-Related Morphological Changes in the Hippocampus of Ts65Dn Mice

**Figure 1:**
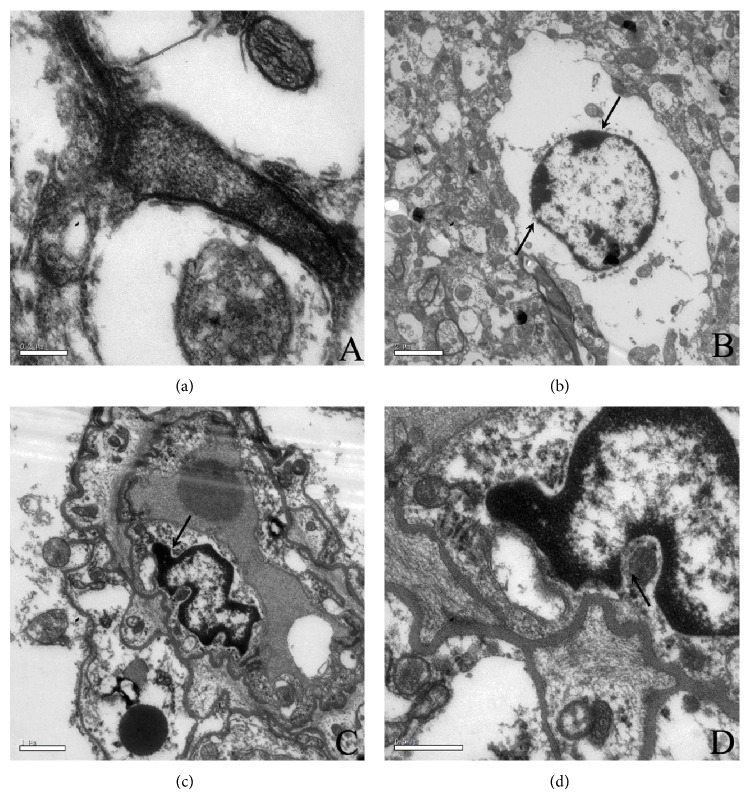
The morphological changes in the hippocampus of Ts65Dn mice observed by TEM. (a) Normal hippocampus tissues in control mice showing typical synaptic and normal cell structure, including normal cell nuclear shapes, distributed chromatin, abundant organelles, dense mitochondria, and flat Golgi apparatus. (b) In Ts65Dn mice the nuclear membrane became thin, chromosomes appeared unevenly distributed, and chromatin crescents were produced. (c) The nuclei exhibited pyknosis and nuclear fragmentation. (d) The cell wall began to sag and cell atrophy was observed.

Typical synaptic structures and normal cell structures were observed in the control mice (Figure 1(a)). However, varying degrees of changes characteristic of apoptosis were observed in the hippocampus of Ts65Dn mice, which mainly included a thinning nuclear membrane, chromosomes that appeared unevenly distributed, chromatin crescents (Figure 1(b)), pyknosis of the nuclei and even nuclear fragmentation was observed. At the same time, the organelles were reduced in size and the mitochondria appeared shrunken (Figure 1(c)). The cell wall sagged, the mitochondria and endoplasmic reticulum were reduced, and autophagic vacuoles had formed (Figure 1(d)).

### 3.2. Apoptosis-Related Gene Expression in the Hippocampus of Ts65Dn Mice

**Figure 2:**
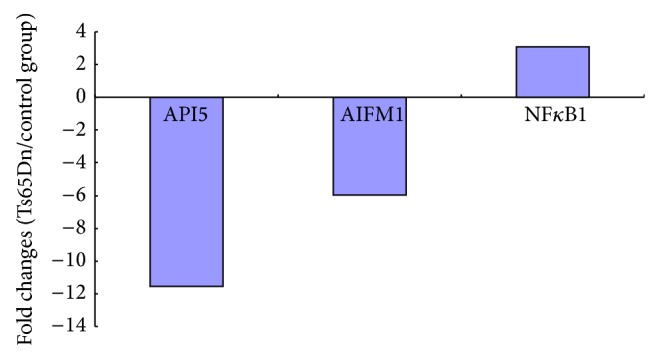
Fold change in gene expression between the Ts65Dn mice and the control group. Compared with the control group, the expression of API5 and AIFM1 showed decreased levels, and NF*κ*B1 expression was increased in the hippocampal tissues of Ts65Dn mice.

**Figure 3:**
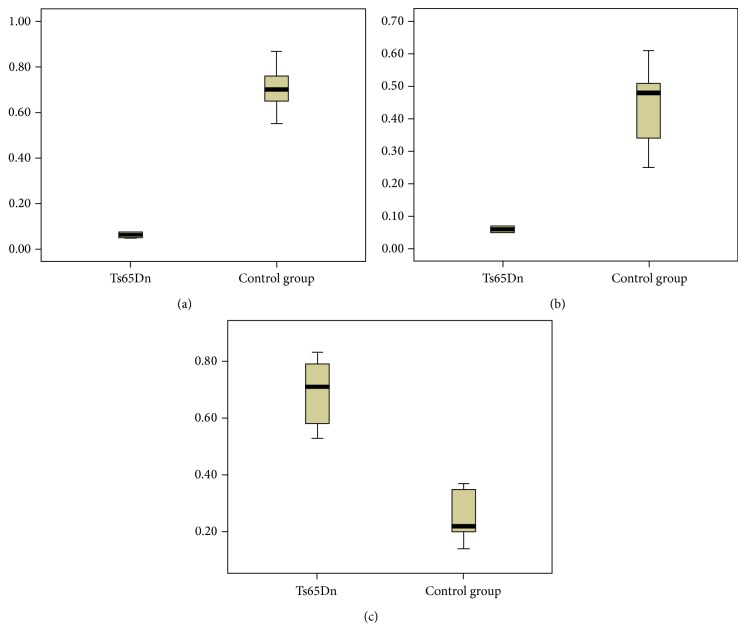
Comparison of the expressions of API5, AIFM1, and NF*κ*B1 by Box plots. Box plots show the comparison of gene expression, calculated by 2-ΔΔCt, in Ts65Dn and the normal control group mice. Data were compared with *t*-tests. (a) API5. (b) AIFM1. (c) NF*κ*B1.

In present study, PCR arrays contained 53 genes related to cell apoptosis. After real-time PCR, three genes (API5, AIFM1, and NF*κ*B1) showed different patterns of expression in the hippocampus of Ts65Dn mice compared to the normal mice (Table 1). Among the three genes, only NF*κ*B1 showed a significant increase in expression, while API5 and AIFM1 showed notably decreased expression. The fold changes of expression of API5, AIFM1, and NF*κ*B1 were 11.55, 5.94, and 3.11, respectively (Figure 2). We compared the significant differences in the expression of API5, AIFM1, and NF*κ*B1 using Box plots, as shown in Figure 3. The gene levels were determined by comparing the value of the fold change in expression (2-ΔΔCt) of the target genes to that of the housekeeping genes in the two groups. We also found significantly different levels of expression in the Ts65Dn mice compared with the normal control group (*P* = 0.00 for API5, *P* = 0.00 for AIFM1, and *P* = 0.02 for NF*κ*B1).

In present study, we examined the genes of the caspase family (including casp-2, casp-3, casp-4, casp-6, casp-7, casp-8, and casp-9). However, they showed no significant differences in expression levels. Meanwhile, several well-known genes related to cell apoptosis, such as Bcl-2, Bad, Bid, Fas, and TNF, also did not differ in expression in the hippocampus of Ts65Dn mice compared to the normal control group (Table 1).

## 4. Discussion

In this study, we showed cell apoptosis in the nervous system of Ts65Dn mice by changes in cell morphology. Then, using a target PCR array, we found that three genes related to cell apoptosis showed different levels of expression in the hippocampus of Ts65Dn mice.

Apoptosis, or programmed cell death, is a regulated physiological process that leads to cell death and is characterized by cell shrinkage, membrane blebbing, and DNA fragmentation. The changes associated with apoptosis often occur in multiple stages. First, there is a decrease in cell volume, and then the cytoplasmic density appears to increase, followed by a decrease in the mitochondrial membrane, an increase in permeability, chromatin condensation, nuclear nucleolar fragmentation, DNA degradation, and finally the formation of apoptotic bodies. In the present study, we also found the typical characteristics of cell apoptosis as stated above. For example, the nuclear membrane thinned, chromosomes appeared unevenly distributed, chromatin crescents were produced, the nuclei exhibited pyknosis, and nuclear fragmentation was observed. It is still uncertain whether cell apoptosis occurs in the brains of DS patients. Through the observation of transmission electron microscopy, we could preliminarily confirm that cell apoptosis occurs in the nervous system of Down syndrome model mice.

Currently, the mechanism of cell apoptosis is not fully understood. However, it is well known that the process is controlled by multiple genes, including the Bcl-2 family, caspase family, TNFR family, C-myc, and P53. In the present study, we included 53 genes in one customized PCR array, including several important genes related to cell apoptosis. For example, we included seven members (casp-2, casp-3, casp-4, casp-6, casp-7, casp-8, and casp-9) of the caspase family in the PCR array. We hoped to discover which gene plays an important role in cell apoptosis of Down syndrome. In contrast to our expectations, all members of the caspase family, including Bcl-2, Bad, Bid, Fas, and TNF, showed normal expression in the hippocampus of Ts65Dn mice compared with the normal control group. Only three genes, API5, AIFM1, and NF*κ*B1, showed altered expression in Ts65Dn mice (with 11.55-, 5.94-, and 3.11-fold changes in expression levels, resp.). Caspase-3 is activated in apoptotic cells both by extrinsic (death ligand) and intrinsic (mitochondrial) pathways [13, 14] and is considered to play a typical role in apoptosis. However, we obtained a negative result for caspase-3 in this study. It is not certain whether the process of cell apoptosis in the nervous system of Down syndrome patients is mediated by caspase-3.

We found that API5, AIFM1, and NF*κ*B1 genes showed significantly altered expression in Ts65Dn mice. They maybe play important roles in apoptosis of Down syndrome patients. API5 encodes the protein apoptosis inhibitor 5, which prevents apoptosis after growth factor deprivation. This protein suppresses the transcription factor E2F1-induced apoptosis and also interacts with and negatively regulates acinus, a nuclear factor involved in apoptotic DNA fragmentation. The consensus view regarding API5 is that it is closely related to cancer and is considered as a target for anticancer drugs [15]. At the same time, its expression is strictly controlled by gene transcription, and NF-*κ*B is the key transcriptional regulator [16]. AIFM1 also encodes an apoptosis-related protein called “apoptosis-inducing factor 1,” which is essential for nuclear disassembly in apoptotic cells and is found in the mitochondrial intermembrane space in healthy cells. Induction of apoptosis results in the translocation of this protein to the nucleus where it affects chromosome condensation and fragmentation. Recently, many studies have focused on the relationship between AIFM1 and nervous system diseases [17]. Interestingly, cell apoptosis induced by AIFM1 is independent of the caspase family [18]. The nuclear factor NF-kappa-B p105 subunit is a protein which, in humans, is encoded by the NF*κ*B1 gene. NF-*κ*B is found in almost all animal cell types and it is formed by p50 and p65, while p50 is encoded by the NF*κ*B1 gene. It is well known that NF-*κ*B plays a key role in regulating the immune response to infection and has been linked to cancer and inflammatory and autoimmune diseases among others. Meanwhile, NF-*κ*B controls the expression of genes which regulate a broad range of biological processes in the central nervous system, and it has also been implicated in processes of synaptic plasticity and memory [19]. It is noteworthy that unbalanced activation of the NF-*κ*B p50/RelA dimer contributes to cell death [20]. Whether it also plays an important role in the neurological impairment of Down syndrome is uncertain which should be the subject of further research.

In conclusion, we found that there were varying degrees of changes characteristic of apoptosis in the hippocampus of Ts65Dn mice. Meanwhile, by PCR array, including several important genes related to cell apoptosis, three genes (API5, AIFM1, and NF*κ*B1) showed changes of expression in the hippocampus of Ts65Dn mice compared with normal mice. However, some well-known genes related to cell apoptosis did not show changes in expression levels. The genes we found which were differentially expressed in the hippocampus of Ts65Dn mice may be closely related to cell apoptosis. PCR array technology can assist in the screening and identification of genes involved in apoptosis.

## Figures and Tables

**Table 1:** Differential apoptotic gene expressions in the hippocampus of Ts65Dn mice.

Gene symbol	Description of genes	Chromosome		Ts65Dn/control group	
		Human	Mouse	Fold changes	*P* value
API5	Apoptosis inhibitor 5	11	2	−11.55	0.00^∗^
AIFM1	Apoptosis-inducing factor, mitochondrion-associated, 1	X	X	−5.94	0.00^∗^
NF*κ*B1	Nuclear factor of kappa light polypeptide gene enhancer in B-cells 1	4	3	3.11	0.02^∗^
Bcl-2	B-cell CLL/lymphoma 2	18	1	2.75	0.07
Ntrk1	Neurotrophic tyrosine kinase, receptor, type 1	1	3	2.59	0.09
Tnfrsf1a	Tumor necrosis factor receptor superfamily, member 1A	12	6	2.52	0.1
Ripk1	Receptor (TNFRSF)-interacting serine-threonine kinase 1	6	13	2.52	0.1
Rela	V-rel avian reticuloendotheliosis viral oncogene homolog A	11	19	2.52	0.1
Map3k14	Mitogen-activated protein kinase kinase kinase 14	17	11	2.52	0.1
Irak1	Interleukin-1 receptor-associated kinase 1	X	X	2.52	0.1
Il1a	Interleukin-1, alpha	2	2	2.52	0.1
Il10	Interleukin 10	1	1	2.52	0.1
Ikbkg	Inhibitor of kappa light polypeptide gene enhancer in B-cells, kinase gamma	X	X	2.52	0.1
Ikbkb	Inhibitor of kappa light polypeptide gene enhancer in B-cells, kinase beta	8	8	2.52	0.1
Igf1r	Insulin-like growth factor 1 receptor	15	7	2.52	0.1
Fadd	Fas (TNFRSF6)-associated via death domain	11	7	2.52	0.1
Dffa	DNA fragmentation factor, 45 kDa, alpha polypeptide	1	4	2.52	0.1
Dapk1	Death-associated protein kinase 1	9	13	2.52	0.1
Cideb	Cell death-inducing DFFA-like effector b	14	14	2.52	0.1
Cidea	Cell death-inducing DFFA-like effector a	18	18	2.52	0.1
Cdc2a	Cyclin-dependent kinase A-1		10	2.52	0.1
Cd70	CD70 molecule	19	17	2.52	0.1
Casp-9	Caspase-9	1	4	2.52	0.1
Casp-8	Caspase-8	2	1	2.52	0.1
Casp-7	Caspase-7	10	19	2.52	0.1
Casp-6	Caspase-6	4	3	2.52	0.1
Casp-3	Caspase-3	4	8	2.52	0.1
Birc2	Baculoviral IAP repeat containing 2	11	9	2.52	0.1
Apaf1	Apoptotic protease-activating factor 1	12	10	2.52	0.1
Akt2	V-akt murine thymoma viral oncogene homolog 2	19	7	2.52	0.1
Akt3	V-akt murine thymoma viral oncogene homolog 3	1	1	3.3	0.13
Il1b	Interleukin-1, beta	2	2	6.75	0.14
Myd88	Myeloid differentiation primary response gene 88	3	9	3.54	0.15
Birc3	Baculoviral IAP repeat containing 3	11	9	3.63	0.15
Mapk3	Mitogen-activated protein kinase 3	16	7	3.21	0.17
Mapk1	Mitogen-activated protein kinase 1	22	16	6.59	0.29
Bid	BH3 interacting domain death agonist	22	6	−2.03	0.3
Fas	Fas cell surface death receptor	10	19	3.23	0.43
Casp-4	Caspase-4	11	9	1.7	0.48
Akt1	V-akt murine thymoma viral oncogene homolog 1	14	12	3.44	0.49
TNF	Tumor necrosis factor	6	17	2.01	0.5
Nf*κ*bia	Nuclear factor of kappa light polypeptide gene enhancer in B-cells inhibitor, alpha	14	12	2	0.56
Cd40lg	CD40 ligand	X	X	1.64	0.61
Pik3r2	Phosphoinositide-3-kinase, regulatory subunit 2	19	8	−1.64	0.72
Casp-2	Caspase-2	7	6	−1.66	0.72
Tnfrsf10b	Tumor necrosis factor receptor superfamily, member 10b	8	14	1.49	0.75
Ppp3cc	Protein phosphatase 3, catalytic subunit, gamma isozyme	8	14	1.58	0.8
Xiap	X-linked inhibitor of apoptosis	X	X	−1.05	0.85
Ppp3r1	Protein phosphatase 3, regulatory subunit B, alpha	2	11	1.22	0.89
Bad	Bcl-2-associated agonist of cell death	11	19	1.15	0.9
Pik3ca	Phosphatidylinositol-4,5-bisphosphate 3-kinase, catalytic subunit alpha	3	3	1.21	0.93
Endog	Endonuclease G	9	2	1.05	0.96
Pik3cg	Phosphatidylinositol-4,5-bisphosphate 3-kinase, catalytic subunit gamma	7	12	−1.01	0.99

Note: ^∗^ *P* < 0.05.
